# Design and Development of a Sensor-Enhanced Remotely Operated Underwater Vehicle (ROUV) Platform for Environmental Monitoring

**DOI:** 10.3390/s26030905

**Published:** 2026-01-30

**Authors:** Dimitrios Tziourtzioumis, George Minos, Triantafyllia Anagnostaki, Eleftherios Kenanidis, Theodoros Kosmanis

**Affiliations:** 1 Laboratory of Energy Systems, Department of Industrial Engineering and Management, School of Engineering, International Hellenic University, 57400 Thessaloniki, Greece; tanagnos@iem.ihu.gr (T.A.); iem2019076@ihu.gr (E.K.); kosmanis@ihu.gr (T.K.); 2 Laboratory of Biology & Histology, Microscopy & Image Analysis, Systematics & Biometry, Department of Nursing, School of Health Sciences, International Hellenic University, 57400 Thessaloniki, Greece; gminos@ihu.gr

**Keywords:** environmental monitoring, remotely operated underwater vehicle, water-quality sensors, aquaculture monitoring

## Abstract

**Highlights:**

**What are the main findings?**

- A modular, sensor-enhanced ROUV platform was successfully designed, integrated, and validated under real aquaculture conditions.
- High-resolution, depth-resolved measurements of temperature, salinity, pH, and dissolved oxygen were obtained using a mobile underwater platform.

**What are the implications of the main findings?**

- Mobile underwater robotic platforms can effectively complement fixed monitoring stations in aquaculture environments.
- The proposed ROUV enables adaptive, localized, and depth-resolved environmental assessment near fish cages.

**Abstract:**

Remotely operated underwater vehicles (ROUVs) have been attracting more attention lately as they are considered to be operationally versatile, capable of real-time communication, and can be fitted with various sensor payloads for environmental monitoring purposes. This study presents the design, development, and field validation of a sensor-enhanced ROUV platform tailored for environmental monitoring and aquaculture applications. The vehicle is equipped with a modular set of sensors for temperature, pH, dissolved oxygen (DO), and electrical conductivity (EC) along with separate signal-conditioning circuits for each sensor and real-time data acquisition from tethered architecture. The general system concept is modularity, reproducibility, and robustness in a marine environment. In situ measurements were performed at an active aquaculture site in the North Aegean Sea throughout several seasons during 2025. Using this system, depth-resolved measurements were obtained with sensor accuracies of ±0.1 °C (temperature), ±0.05 pH units, ±0.05 mg/L (dissolved oxygen), and ±2% (electrical conductivity). The following sections describe the development and aquaculture testing of the platform, which yielded stable and repeatable operation in real conditions.

## 1. Introduction

Aquatic ecosystems have undergone significant changes over the last few decades because of industrialization, urbanization, agricultural runoffs, and climate change. These changes have led to more pollution, eutrophication, habitat destruction, and the extinction of many native species [1]. The global ocean environment is getting harder to deal with, which is why there is a growing need for complete monitoring systems that can keep an eye on the ocean all the time in a changing and often dangerous environment [2].

Ship-based sampling, manual diving operations, and stationary sensors, such as buoys and moored instruments, are the most common ways to keep an eye on underwater ecosystems [3]. These methods have been very useful in oceanography and limnology, but they are often limited by high costs, low spatial coverage, and safety concerns with manual diving [4]. Diver-based sampling methods are not very good at quickly sampling shallow areas or overtime. Fixed sensor station systems, on the other hand, give stationary measurements from a certain area or zone. They cannot be moved to the same place to see how the weather changes. The small number of samples taken at certain times may also make it impossible to find temporary or localized environmental conditions [5].

Besides their great flexibility, underwater robotic systems, especially Remotely Operated Underwater Vehicles (ROUVs), are a great alternative for environmental monitoring. Making environmental measurements more localized and adaptive, ROUVs offer the advantages of real-time data transmission, precise maneuvering, and direct operator supervision [6,7,8]. In terms of environmental monitoring, ROUVs are generally a more versatile tool than Autonomous Underwater Vehicles (AUVs). While AUVs can carry out long-distance, pre-programmed tasks, they might need advanced navigation systems and extra onboard computing and planning, thus making them more complicated to operate than ROUVs [9]. Since ROUVs have simple operational architecture, they can be operated under direct operator supervision with real-time feedback. This feature increases mission reliability and, consequently, reduces the risk of losing valuable sensor data in an inherently uncertain environment. Furthermore, ROUVs can continuously transmit real-time sensor data and video images back to the surface control station for analysis [10,11].

Improved environmental monitoring capabilities are one of the major benefits of adding more advanced sensor technologies to ROUVs [12]. The use of such sensing devices allows for on-site measurement of most important physicochemical and biological parameters, such as temperature, pressure, electrical conductivity, salinity, pH, dissolved oxygen, turbidity, chlorophyll concentration, and presence of pollutants in the aquatic environment [13]. These parameters are very important for assessing the quality of water, the health of the ecosystem, and the extent of human influence on the environment [14]. Most environmental sensors include high-resolution sonar and navigation systems. This enables the ROUV to generate Geographic Information System (GIS) data and provide environmental modeling and impact assessments with extensive data [15].

The design and development of sensor-equipped real-time operation features and autonomous underwater vehicle technologies involve many technical and operational difficulties [16,17,18]. A sensor-equipped advanced underwater vehicle must be strong and robust to withstand very severe hydrodynamic stresses and seawater corrosion, avoid being overgrown by marine organisms, and tolerate temperature changes without its waterproof and reliable nature being compromised [19]. The operation of these vehicles is to a large extent dependent on embedded systems which are capable of efficiently collecting real-time sensor data, integrating multiple sensor sources, and communicating that information to a surface control station. A further significant design feature is the platform’s versatility and upgradability that allows the system to be reconfigured for different environmental monitoring tasks without the necessity of changing the main hardware components [15,20,21,22].

Recent studies have shown that low-cost modular underwater robots are an increasing focus of research on the use of ROUVs [23]. The advent of microcontrollers, single-board computers, and compact sensors, combined with open-source hardware and software frameworks, has reduced barriers to entry for creating custom ROUV systems from scratch [6,7]. Thus, ROUVs have begun to support an increasing number of small-scale research projects and community-based monitoring and research. Still, most existing platforms are built primarily for specific applications, with no common means of integrating sensors into their designs and little to no adherence to established metrics for long-term reliability and data quality [24]. Developing a remotely operated environmental monitoring underwater vehicle equipped with various sensors is of the highest priority. The platform is designed to achieve high performance, low cost, real-time data acquisition, and versatility while offering a robust and easy-to-navigate design [25,26]. Unlike many existing platforms, the proposed ROUV was validated through multi-season field deployment in an active aquaculture facility.

This paper is organized as follows: Section 1 provides an overview of previous research on the application of underwater robotics systems to environmental monitoring, as well as an overview of prior research on ROUVs with enhanced sensors for specific purposes. Section 2 describes the mechanical, electrical, and software architecture of the developed ROUV platform. Section 3 presents the experimental results obtained from the field deployments, followed by their interpretation in Section 4. Conclusions and future directions are summarized in Section 5.

Inspired by these shortcomings, this research is mainly about conceptualizing and validating by experiments a modular ROUV-based environmental sensing platform. Even though commercial and research-oriented ROUV platforms are increasingly at the disposal of users, the majority of present-day systems are one application only, are not equipped for modular sensor integration, or have very little validation under real operational conditions. In this paper, a sensor-augmented ROUV is built which prioritizes modularity, scalability, and reproducible calibration procedures for multi-parameter environmental monitoring. The system presented here features a compact sensor cluster that not only measures temperature, pH, DO, and EC simultaneously but also ensures the stable operation of the vehicle and supports real-time data transmission via a tethered architecture. Additionally, the platform offered here is not a mere set of devices used in laboratory testing or focusing on single-parameter sensing that serves the purpose of intriguing articles, but it is field-proven through deployment in an aquaculture installation.

The main elements of this work include: (i) the installation of a sensor cluster within a compact ROUV framework; (ii) the development and implementation of a data acquisition system suitable for real-time environmental monitoring; and (iii) experimental validation demonstrating the system’s suitability to real aquaculture case study.

## 2. Materials and Methods

### 2.1. Technical Description of the ROUV

The BlueROV2 chassis (Blue Robotics Inc., Torrance, CA, USA) served as the base platform for integrating the developed sensor cluster apparatus. The ROUV is powered through a buoyant tether that is well-suited for submerged operations, offering strength, durability, and good electrical characteristics because of a Kevlar-strengthened core surrounded by a polyurethane jacketed cable [27].

In terms of the mechanical design of the ROUV, the design contains flotation boxes, pressure vessels, and water-tight compartments that hold the control circuits and the camera. In that respect, the ROUV is designed with support for four T200 vertical thrusters and four T200 vectored thrusters. In addition to that, the ROUV is designed with two LED lights that enhance underwater visibility when operating.

**Figure 1:**
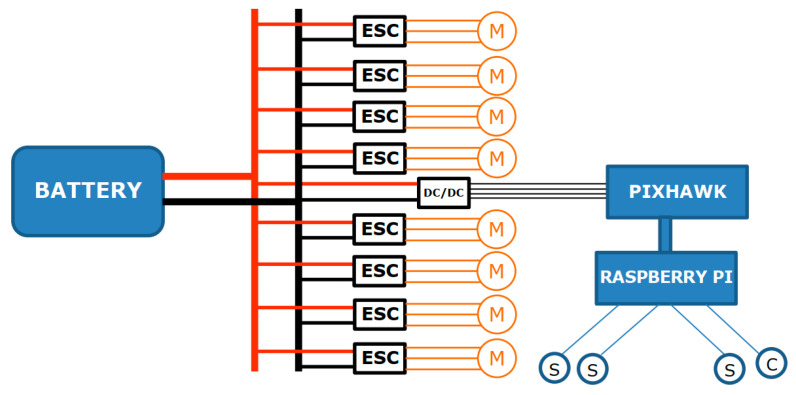
Schematic diagram of the Remotely Operated Underwater Vehicle power system, including thruster motors (M), sensor probes (S), camera (C), and electronic speed controllers (ESC).

While the mechanical structure enables stable and efficient underwater motion, the electrical and electronic systems govern all vehicle operations and overall functionality. From a system-level perspective, electrical architecture can be divided into two main subsystems: power and control subsystems. The power subsystem comprises the energy source and all components with significant power consumption, including the eight thrusters, two LED lights, and the sensor cluster apparatus. The ROUV is powered by a 4S lithium-ion battery with a nominal voltage of 14.8 V and a capacity of 15.6 Ah. A schematic representation of the main power system configuration used during field measurements is shown in Figure 1.

### 2.2. Sensor System for Environmental Monitoring

A modular sensor system has been designed to carry out the measurement of key water quality parameters that are essential for aquaculture. The system is equipped with multiple sensors, one of which is for pH measurement using an Atlas Scientific ENV-50-pH sensor (Atlas Scientific, Long Island, NY, USA). The second sensor measures the electrical conductivity of seawater with an Atlas Scientific ENV-40-EC-K10 sensor (Atlas Scientific, USA), and from this, total dissolved solids (TDS) and salinity were derived using standard formulae. The concentration of dissolved oxygen (DO) was determined by an Atlas Scientific ENV-50-DO sensor (Altas Scientific, USA), which is very accurate and capable of making oxygen measurements in marine environments.

Temperature compensation is taken care of inside the EZO™ interface circuits with the help of real-time temperature input from the Celsius sensor; thus the pH, dissolved oxygen, and electrical conductivity measurement values are being automatically corrected for the temperature-dependent effects.

The water temperature measurement used a “Celsius Fast-response” thermal sensor with high accuracy (Blue Robotics Inc., USA) which was mounted outside the watertight case of the ROUV to allow the sensor to be in direct contact with the surrounding water and measure water temperatures straight away. Parameters like temperature, pH, salinity, and dissolved oxygen were previously discovered as important factors used to judge water quality by various studies [28,29,30].

All sensors were connected through EZO™ signal conditioning circuits (Atlas Scientific, USA), i.e., EZO-pH, EZO-DO, and EZO-EC, which besides signal conditioning also perform temperature compensation and enable digital communication with the onboard controller. The EZO circuits were set up for digital communication and calibrated following the manufacturer’s instructions prior to the field deployment. The major technical specifications of the sensors used, such as measurement ranges and accuracies, are given in Table 1.

All sensors and interface circuits were chosen from a marine environment series, and they were also compatible with the embedded control architecture of the ROUV.

The selection of sensor measurement ranges and accuracies relied on normal environmental conditions that could be encountered in coastal Mediterranean aquaculture systems. The bounds of seawater temperature (10–30 °C), salinity (35–39 psu), pH (7.8–8.3), and dissolved oxygen (4–10 mg/L) used as reference ensured that all the sensors that were selected would be performing well within their optimal measurement ranges. The accuracies of the sensors were decided such that they would be greater than the minimum resolution needed to detect environmentally relevant gradients and variability at very short time intervals, at the same time, being robust and stable over the long-term in marine conditions.

Temperature compensation is handled internally by the EZO™ interface circuits using real-time temperature input from the Celsius sensor, ensuring that pH, dissolved oxygen, and electrical conductivity measurements are automatically corrected for temperature-dependent effects.

Regarding measurement accuracy, for standard BlueROV operations, pressure sensors combined with ArduSub software (Blue Robotics Inc., Torrance, CA, USA, v.4.0.3) provide reliable depth control. The depth uncertainty is now presented in Table 1. Specifically, according to the manufacturer’s specifications for the ROUV pressure (barometric), sensor Bar30 offers good precision (depth resolution of 2 mm).

**Figure 2:**
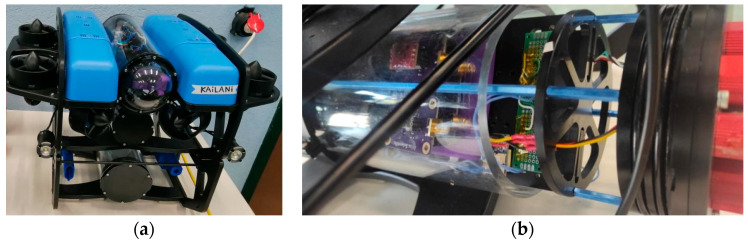
(**a**) Assembled view of the ROUV, including the sensor electronics vessel mounted at the bottom of the chassis; (**b**) close-up view of the sensor electronics vessel.

The electronics of the sensor cluster are housed in a waterproof cylindrical enclosure mounted to the bottom of the ROUV chassis. The sensor probes were installed using custom-designed, 3D-printed mounting bases. An overview of the ROUV used for this study’s measurements is shown in Figure 2. On the left side of Figure 2 an assembled view of the ROUV is presented, including the sensor electronics vessel at the bottom of the chassis, the battery vessel at the middle level of the chassis, the main electronics vessel at the top level of the chassis, the lights, four vertical thrusters, and four vectored thrusters. On the right side, a close-up view of the sensor electronics vessel including sensor carried boards is shown.

**Figure 3:**
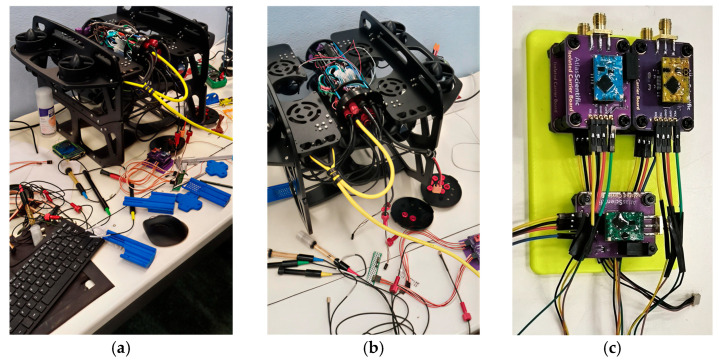
(**a**) Exploded view of the ROUV sensor electronics vessel, illustrating the connection of the sensor cables to the Raspberry Pi; (**b**) close-up view of the top vessel of the ROUV; (**c**) 3D printed mounting base for the sensor carrier board.

The installations as well as the integration process of the sensors’ electronics on the ROUV together with the associated ROUV mounting bases are illustrated in Figure 3. The sensor mounting bases were 3D printed using a Bambu Lab P1S printer (Bambu Lab, Shenzhen, China) with ABS (Shenzhen Esun Industrial Co., Ltd., Shenzhen, China) filament, chosen because of its mechanical strength and compatibility with underwater deployment.

**Figure 4:**
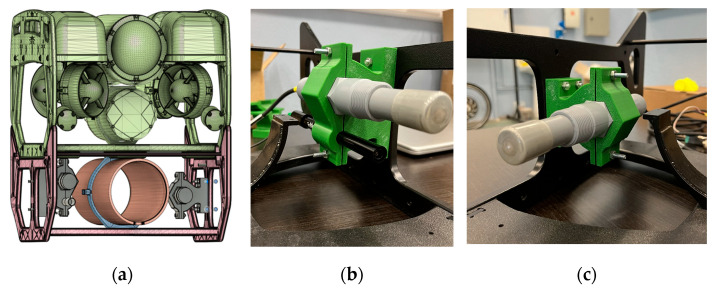
(**a**) Three-dimensional view of the ROUV, including all additional components; (**b**) 3D-printed dual sensor mounting base; (**c**) 3D-printed single sensor mounting base.

As the sensor mounting bases were going through the last design phase, a 3D model of the ROUV with the chassis extensions and the additional cylindrical vessel was made in Autodesk Fusion 360 (Autodesk, Inc., San Fransico, CA, USA). Once the design was finished, the sensor mounting bases were 3D-printed and fixed on the ROUV chassis. This method is shown in Figure 4, which depicts a 3D view of the ROUV along with the sensor mounting bases. The two designs shown in Figure 4b,c are different sensor mount designs. Figure 4b displays two different types of sensor mounting base, e.g., dissolved oxygen (DO) and electrical conductivity (EC) are the two types of sensor mounts shown. In addition, in Figure 4c, one type of mounting base sensor from single pH is shown.

Prior to deployment, protective caps were removed, pH and DO probes were stored in manufacturer-recommended wet storage solutions, and all sensors were rinsed with deionized water after recovery to ensure measurement stability and prevent contamination.

### 2.3. Sensor Calibration Process

Calibration of all environmental sensors was done before a field deployment by following the manufacturer’s instructions. The pH sensor was calibrated by a three-point calibration method (pH 4, 7, and 10). The electrical conductivity was calibrated with two standard solutions that are suitable for the K10 probe. Dissolved oxygen was calibrated with a two-point method that was based on air-saturated conditions and a zero-oxygen reference.

The temperature and pressure sensors provided by Blue Robotics came factory-calibrated and were rated for direct marine usage; hence, the user did not do any additional calibration. Subsequent to calibration, the functionality of all sensors has been checked to be within their specified accuracy ranges.

### 2.4. ROUV Control Subsystem, Sensor Integration, and Data Connectivity

The control subsystem of an ROUV refers to the exchange of information between various onboard electronic components and the surface control station. The latter consists of a computer equipped with dedicated control and monitoring software and is connected to the ROUV via a tether. The control subsystem manages the actuation of the vehicle, the acquisition of sensor data, as well as the communication that takes place from the underwater platform to the surface operator.

**Figure 5:**
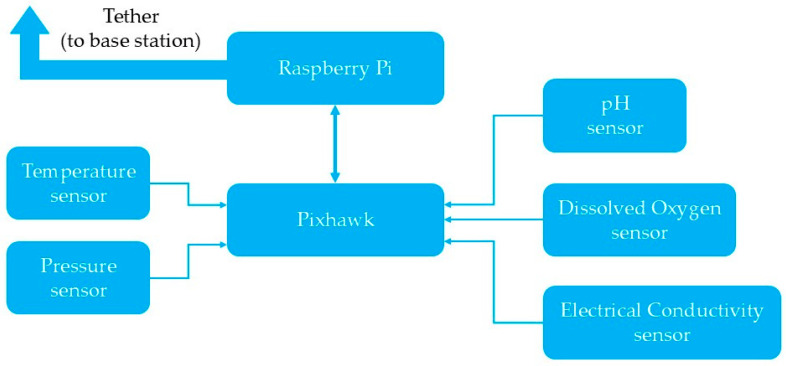
Block diagram of the ROUV control subsystem showing sensors, Raspberry Pi, and Pixhawk used in environmental monitoring.

For the hardware side, the control subsystem consists of controllers, sensors, actuator control circuits, and a tether which allows the communication between the control station and the ROUV. The block diagram depicting the control system with the sensor and the two processing units, Raspberry Pi 3 Model B and Pixhawk, is shown in Figure 5. The list of software for Raspberry Pi 3b and Pixhawk 2.4.8 PX4 includes the following: Raspberry Pi OS (Raspberry Pi Ltd., Cambridge, UK, v.13) Python (Python Software Foundation, Wilmington, DE, USA, v.3.14.0), MAVLink (Auterion, Arlington, VA, USA, v.2.0) and QGroundControl (Auterion, Arlington, VA, USA, v.5.0.8). Furthermore, it should be noted that data fusion is not applied, as the sensors operate independently, and processed values (pH, EC, DO, temperature) are displayed in real time on a custom GUI at the surface station.

The Pixhawk autopilot handles low-level vehicle control, including thruster actuation and stabilization. The Raspberry Pi (RPi) is a companion computer that helps with managing sensors, processing data, and talking to the surface control station. Although the Pixhawk supports limited sensor interfacing, its computational resources are constrained. Therefore, the RPi acts as an intermediary, handling high-level data acquisition and bridging communication between the sensors and the surface computer.

**Figure 6:**
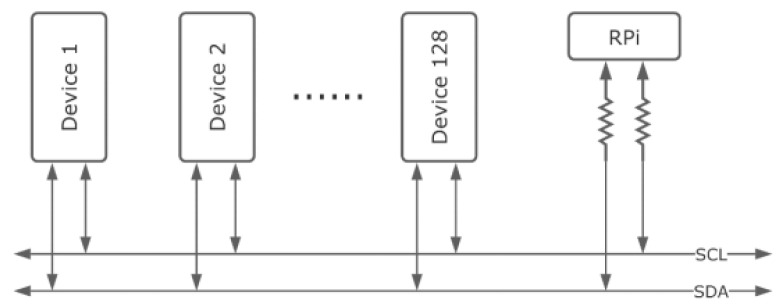
Simplified diagram of the I^2^C communication bus showing the serial data (SDA) and serial clock (SCL) lines shared between the master device and multiple sensor nodes.

Sensor integration with the RPi is achieved through standard digital communication protocols, including Inter-Integrated Circuit (I^2^C), Serial Peripheral Interface (SPI), and Universal Asynchronous Receiver–Transmitter (UART). Because the RPi lacks integrated analog-to-digital and digital-to-analog converters, sensors cannot be connected directly in analog form. Instead, dedicated interface boards digitize sensor outputs and serialize the data. The I^2^C protocol was chosen from the available options because it is simple, allows multiple devices to share a bus, and is good for observation-class ROUVs, like the one built in this study. A shared I^2^C bus was set up and connected to each sensor that was already in use. The two signal wires in the bus include the serial data wire (SDA) and the serial clock wire (SCL). The resistors are used to pull up the wires, and the value of the resistors to be used depends on the length of the bus. A simplified diagram showing the architecture of the I^2^C bus is depicted in Figure 6. As a synchronous protocol, all sensor communications are synchronized to the clock signal generated by the master device. The protocol supports multiple data rates, from 100 kbps to several Mbps, depending on the bus’s physical characteristics.

A representative sensor interfacing scheme is shown in Figure 6, which illustrates the typical connection topology between the Raspberry Pi and the EZO™ sensor interface boards. Each sensor probe is connected to its corresponding EZO™ circuit, which performs signal conditioning, temperature compensation, and analog-to-digital conversion before transmitting calibrated digital data via the I^2^C bus. This architecture is identical for pH, dissolved oxygen, electrical conductivity, and pressure sensors, ensuring consistency and modularity across the sensing subsystem.

Every device connected to the I^2^C bus has a unique address that is incorporated into every transaction. Transactions occur in a predictable manner: start condition, address frame, read/write bit, signals for acknowledgment, data frames, and stop condition. All the devices shared common ground, and to make them more interface friendly with a Raspberry Pi, they are also equipped with compatible voltage levels.

The sensor interface boards are responsible for signal conditioning, digitization, and serialization of the signal before it is transmitted to the RPi. All processes of data requests, message decoding, as well as polling of the sensor interfaces are conducted by special scripts running on the RPi. The acquired sensor data are either stored locally on the RPi or transmitted to the surface computer via the Secure Shell (SSH) protocol. At the surface control station, data are accessed and visualized using a custom application that runs alongside the main ROUV control software, QGroundControl (QGroundControl Development Team).

**Figure 7:**
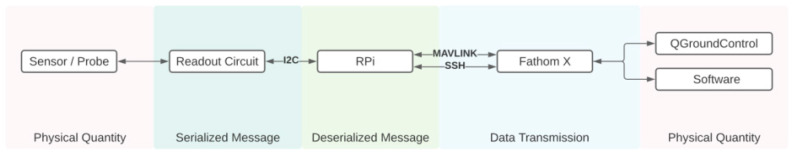
Software and data acquisition architecture presenting sensor data transfer via the I^2^C bus to the Raspberry Pi and subsequent transmission to the surface computer.

The developed software architecture can be divided into two major components as highlighted in Figure 7. These components are the RPi side, which has a script that runs and fetches data from the sensors, and the program that performs the same operations on the surface computer. A graphical user interface (GUI) has been developed as part of the implementation on the surface computer to aid in monitoring and managing the sensor’s information. These two software components have been designed to run concurrently using threads. This approach has been taken to prevent high CPU use and ensure the efficiency of the system.

**Figure 8:**
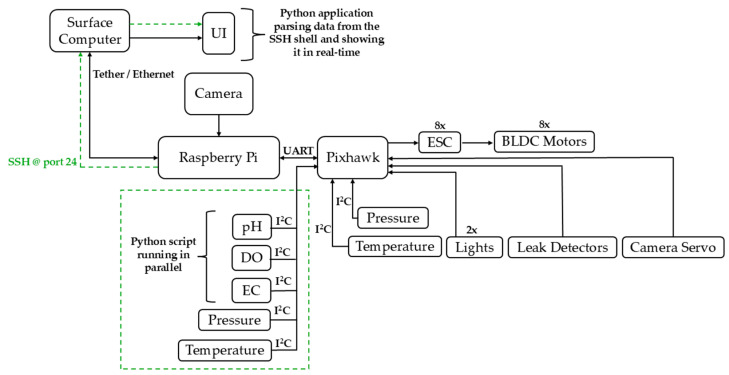
Overview of the complete ROUV system architecture, including onboard components, sensors, actuators, and communication links between the underwater platform and the surface control station.

In summary, Figure 8 presents a complete overview of the ROUV control subsystem including onboard components, sensors, actuators, communication links between the underwater platform and the surface computer.

### 2.5. Field Measurement Procedure

**Figure 9:**
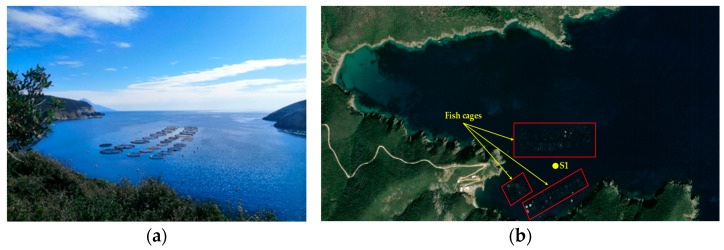
Overview of the study area and measurement location: (**a**) top view of the aquaculture facility; (**b**) satellite view [36] of the gulf showing the location of the fish cages (red shapes) and the measurement point (Station 1, yellow circle).

Field measurements were conducted at an active aquaculture facility in the North Aegean Sea during five campaigns in 2025 (February, April, July, September, and December), thus covering all climatic seasons. The measurements were carried out at a single point of the monitoring station situated at the center of the fish-cage array (39°57′35.3″ N, 23°58′54.5″ E) (Figure 9).

Measurements were made at four depths: 0.3, 5, 10, and 15 m. The sampling frequency of 1 Hz was decided as a compromise between the following: capturing short-term sensor fluctuations, ensuring stable averaging under slowly varying environmental conditions and avoiding unnecessary data redundancy and communication overhead during tethered operation. At each depth, data were recorded for 60 s and repeated three times. Measurement parameters are shown in Table 2.

The monitored water quality parameters were temperature (°C), pH, salinity (psu), and dissolved oxygen (mg/L).

## 3. Results

**Figure 10:**
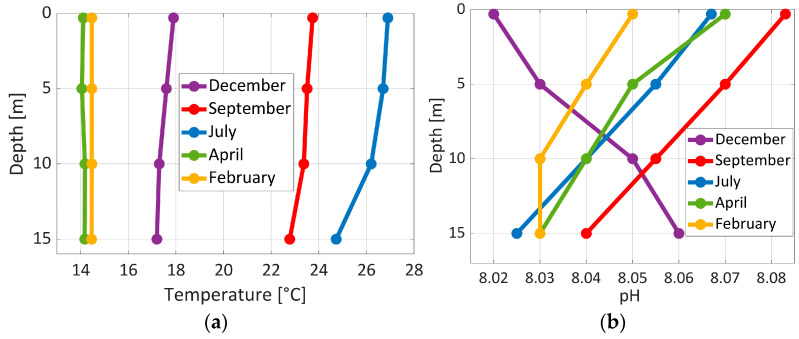
Distribution of (**a**) temperature and (**b**) pH profiles as a function of depth at Station 1 from February to December 2025.

**Figure 11:**
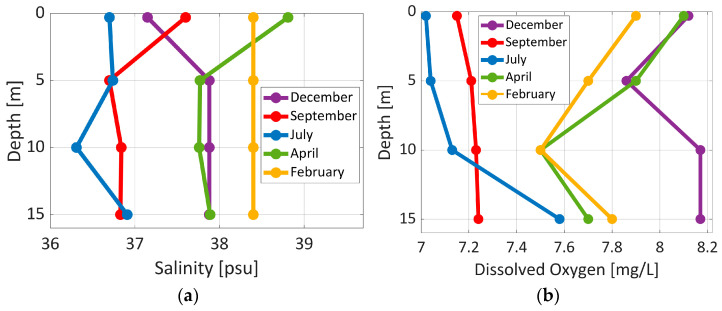
Distribution of (**a**) salinity and (**b**) dissolved oxygen profiles at Station 1 from February to December 2025.

Representative depth profiles of environmental parameters (temperature, pH, conductivity/salinity, and dissolved oxygen) are shown in Figure 10 and Figure 11. As the field measurements are observational rather than controlled data, depth is used as the independent reference variable or ordinate. Whereas the environmental parameters represent the measured environmental responses at each depth.

The temperature profiles reveal well-mixed conditions during spring and winter, while during summer months the water is more stratified. pH values were consistent with the expected Mediterranean range throughout the measurement period. The largest temperature differences between the surface and the sea bottom were about 8 °C at different times of the year. Unlike temperature, salinity showed only slight seasonal variation. Dissolved oxygen, on the other hand, showed a larger degree of variability due to temperature changes and biological activity.

Figure 10 shows that the temperature profiles changed with the seasons. In April, temperatures that were about 14 °C all the way up and down the water column showed that the water was well-mixed, which is common in the spring. In July, the hottest temperatures were near the surface, and they seemed to get cooler as the depth increased. This shows strong stratification during summer months. In September, the temperatures stayed high, but the vertical gradients got smaller. This suggests that autumn mixing was starting. By December, the temperatures had dropped a lot and were almost the same at all heights, which is what you would expect when mixing conditions are fully restored.

In addition, Figure 10 shows that the measured pH values stayed within the expected Mediterranean range (about 8.0–8.1) all year long. The spring and late autumn profiles had minimal vertical variation, indicating that the conditions were well-mixed. In the summer and early fall, however, weak vertical pH gradients were exhibited, with values at depth a little lower. This pattern is in concert with less photosynthesis and more respiration occurring beneath the surface layer.

Differences in the distribution of salinity ranged from about 36 to 38 psu across different depths and months (see Figure 11). When compared to temperature, salinity has shown less variation. Spring and late autumn vertical profiles were uniform, while summer and early autumn conditions revealed slightly fresher values. The lower salinity with respect to the standard open Aegean Sea signals the effect of the local coastal circulation along with the intrusion of Black Sea Water in the North Aegean Sea.

Dissolved oxygen varied significantly throughout the year as presented in Figure 11. Maximum concentrations occurred in April when water temperature is low and oxygen solubility is high under well-mixed conditions. In July and September, oxygen levels were diminished, with minor increases at depth linked to more tranquil subsurface waters. In December, dissolved oxygen levels were comparatively low across the water column, with a slight increase at greater depths, indicating heightened biological oxygen demand near the aquaculture facility in late autumn.

At each measurement depth, sensor data were recorded at a frequency of 1 Hz for 60 s and repeated three times. The values presented in the figures correspond to averaged measurements, providing an assessment of short-term repeatability rather than long-term temporal variability.

## 4. Discussion

The present study does not aim to provide a statistically exhaustive assessment of seasonal variability. Instead, it demonstrates the technical capability of the proposed ROUV platform to acquire repeatable, depth-resolved measurements of key water quality parameters under real operational conditions. Each data point presented in the vertical profiles represents the mean of three repeated measurements acquired over a 60 s interval at a fixed depth. While the limited temporal and spatial sampling preclude definitive conclusions regarding seasonal ecosystem dynamics, the measured trends are consistent with published observations for the region and confirm the system’s suitability for targeted, short-term environmental investigations.

In order to understand the significance of the acquired data, the observed vertical structures were found to be in line with the extensively studied hydrographic and biogeochemical processes in the North Aegean Sea. Several studies which utilized long-term buoy data and ship-based observations have indicated that seasonal temperature-driven stratification is the main factor determining the water column in this area. These studies have also reported well-mixed conditions in winter and spring and strong temperature layering in the summer months [37,38].

The variations in salinity in the North Aegean Sea are generally less than those in temperature and primarily depend on the circulation of the region and the sporadic inflows of Black Sea Water, as have been revealed by observational and modeling studies on a basin scale [38,39]. The patterns of dissolved oxygen in the coastal and shelf areas of the Aegean Sea have been demonstrated to illustrate the combined effects of thermal stratification, oxygen solubility, and biological processes such as respiration and decomposition of organic matter, especially in areas under the influence of fish farming [35,37].

The depth-resolved patterns measured by the proposed ROUV platform are in qualitative agreement with these established regional characteristics, as well as with near-real-time products provided by operational monitoring systems such as the POSEIDON network and the Copernicus Marine Service [39,40]. This agreement supports the reliability of the sensor integration, calibration procedures, and data acquisition strategy employed in the present study, while emphasizing that the primary contribution of this work lies in the validation of a mobile, modular monitoring platform rather than in a comprehensive analysis of regional seasonal dynamics.

Temperature was found to be the major factor controlling seasonal stratification, with well-mixed conditions in spring and autumn and strong temperature stratification in summer. Temperature stratification exerts strong control on pH, salinity, and dissolved oxygen profiles with depth and emphasizes the need to obtain vertical profiles. The constant pH values around the typical Mediterranean value and the absence of vertical salinity gradients indicate that density stratification is temperature-controlled at the measurement site.

Among the parameters measured, dissolved oxygen showed the greatest variation that correlates with the combined effects of thermal stratification, oxygen solubility, and biological processes. The ROUV platform’s sensitivity in detecting the micro-vertical differences in parameters crucial for aquaculture operations suggests its potential use in such applications.

Comparison with previous studies conducted in the North Aegean Sea, including real-time buoy measurements of seasonal temperature and salinity variability [37] and analyses of the basin’s thermohaline structure [38], together with data and visualization tools provided by the Poseidon system of the Hellenic Centre for Marine Research [39] and the Copernicus Marine Service [40], confirms that the observed hydrographic and biogeochemical patterns observed in this study are consistent with earlier investigations. These investigations indicate: intense stratification related to temperature in summer, increased vertical mixing during spring and autumn, and moderate salinity variability driven by local circulation and Black Sea water inflows. Similar seasonal variability of dissolved oxygen, with increased concentrations during mixed periods and lowered concentrations during periods of stratification, has been detected in coastal and shelf regions. Consistency between experimental results in this study and previous work strongly supports the sensor integration, calibration procedures, and data acquisition strategy carried out on the ROUV platform as highly reliable.

From an aquaculture management point of view, the ability demonstrated to retrieve high-resolution vertical profiles conveys several valuable lessons on the assessment of environmental conditions around fish cages, especially during thermal stratification and low levels of dissolved oxygen. The ROUV’s aptitude for mobility and maneuverability enables in situ targeted measurement that is hard to obtain from fixed or surface-based monitoring systems, hence supporting adaptive management and fully informed decision-making.

While the present dataset is not intended to provide a comprehensive characterization of seasonal ecosystem dynamics, it illustrates the platform’s suitability for short-term, localized investigations and targeted environmental assessments near fish cages. The modular design allows rapid deployment and reconfiguration, making the system particularly useful for operational monitoring and diagnostic surveys.

Limitations of the present study include the single monitoring location and relatively short deployment durations. Long-term monitoring, sensor drift assessment, and multi-station surveys will be addressed in future work.

In summary, the data demonstrates the ability of the sensorized ROUV system tested in the field to resolve the hydrographical features and related water quality variations in a complex environment. High vertical resolution data are valuable in understanding the interactions between physical forcing and biogeochemical-mediated responses to provide a basis for the use of mobile underwater sensor systems in environmental mapping around aquaculture facilities.

## 5. Conclusions

This work demonstrated how a modular, sensor-enhanced ROUV platform was designed, integrated, and successfully field-deployed in aquatic environmental monitoring. The system proved capable of acquiring reliable, depth-resolved measurements of temperature, salinity, pH, and dissolved oxygen in an active aquaculture environment under real operational conditions.

These results verify that the proposed platform is particularly suited for localized, short-term investigations and fast assessments of the environment close to fish cages, supplementing any fixed or surface-based monitoring systems. While the system architecture allows for future expansion, long-term autonomous monitoring, sensor longevity, and calibration drift were beyond the scope of the present study and will be addressed in future work through extended deployments, multi-station surveys, and systematic durability assessments.

## Figures and Tables

**Table 1:** Main technical specifications of the environmental monitoring system. The sensor accuracy values were taken from the manufacturer’s specifications [31,32,33,34,35].

Sensor Model	Measured Parameter	Measuring Range	Accuracy
Blue Robotics Bar High-Resolution Depth/Pressure	Depth Pressure	0–295.6 m 0–30 bar	0–45 °C ± 200 mbar (204 cm)
Blue Robotics Celsius Fast-Response	Temperature	−40–125 °C	±0.1 °C
Atlas Scientific ENV-50-pH	pH	0–14	±0.05
Atlas Scientific ENV-50-DO	Dissolved Oxygen	0−100 mg/L	±0.05 mg/L
Atlas Scientific ENV-40-EC-K10	Electrical Conductivity	10 μS/cm–1 S/cm	±2%

**Table 2:** Measurement depths, parameters and acquisition protocol.

Depth (m)	Parameters	Sampling Frequency (Hz)	Duration (s)	Repetitions
0.3	T, DO, S, pH	1	60	3
5	T, DO, S, pH	1	60	3
10	T, DO, S, pH	1	60	3
15	T, DO, S, pH	1	60	3

## Data Availability

The data presented in this study are available from the corresponding author upon reasonable request.

## Abbreviations

The following abbreviations are used in this manuscript:

ROUV	Remotely Operated Underwater Vehicle
AUV	Autonomous Underwater Vehicle
ABS	Acrylonitrile–Butadiene–Styrene
DO	Dissolved Oxygen
EC	Electrical Conductivity
RPi	Raspberry Pi
I^2^C	Inter-Integrated Circuit
SPI	Serial Peripheral Interface
UART	Universal Asynchronous Receiver–Transmitter
SDA	Serial Data Line
SCL	Serial Clock Line
SSH	Secure Shell
GIS	Geographic Information System
